# China’s air quality improvement strategy may already be having a positive effect: evidence based on health risk assessment

**DOI:** 10.3389/fpubh.2023.1250572

**Published:** 2023-10-10

**Authors:** Xianmang Xu, Wen Zhang, Xiaofeng Shi, Zhi Su, Wei Cheng, Yinuo Wei, He Ma, Tinglong Li, Zhenhua Wang

**Affiliations:** ^1^Heze Branch, Biological Engineering Technology Innovation Center of Shandong Province, Qilu University of Technology (Shandong Academy of Sciences), Heze, China; ^2^Shandong Analysis and Test Center, Qilu University of Technology (Shandong Academy of Sciences), Jinan, China; ^3^Shanghai Key Laboratory of Atmospheric Particle Pollution and Prevention (LAP3), Department of Environmental Science and Engineering, Institute of Atmospheric Sciences, Fudan University, Shanghai, China; ^4^Department of Clinical Medicine, Heze Medical College, Heze, China; ^5^Heze Ecological Environment Monitoring Center of Shandong Province, Heze, China

**Keywords:** health risk, economic loss, PM_2.5_ pollution, exposure, prevention and control strategies

## Abstract

Aiming to investigate the health risk impact of PM_2.5_ pollution on a heavily populated province of China. The exposure response function was used to assess the health risk of PM_2.5_ pollution. Results shows that the total number of premature deaths and diseases related to PM_2.5_ pollution in Shandong might reach 159.8 thousand people based on the new WHO (2021) standards. The health effects of PM_2.5_ pollution were more severe in men than in women. Five of the 16 cities in Shandong had higher health risks caused by PM_2.5_ pollution, including LinYi, HeZe, JiNing, JiNan, and WeiFang. PM_2.5_ pollution resulted in nearly 7.4 billions dollars in healthy economic cost, which accounted for 0.57% of GDP in Shandong in 2021. HeZe, LiaoCheng, ZaoZhuang, and LinYi were the cities where the health economic loss was more than 1% of the local GDP, accounted for 1.30, 1.26, 1.08, and 1.04%. Although the more rigorous assessment criteria, the baseline concentration was lowered by 30 μg/m^3^ compared to our previous study, there was no significant increase in health risks and economic losses. China’s air quality improvement strategy may already be having a positive effect.

## Introduction

Air pollution and population health have always been hot topics in the field of environmental research (1–4). In the past decades, air pollution has caused a series of serious health hazards to people in China (5–8). As one of the main pollutants of air pollution, fine particulate matters (PM_2.5_) contains complex chemical components which including various toxic substances (9–11). Because of its diminutive size, PM_2.5_ can enter in the respiratory tract and lungs (12). Once some toxic substances enter the human bloodstream, they may increase the burden on the heart (13). Long-term exposure to high concentrations of PM_2.5_ will increase the health risk of the population, especially the respiratory diseases and cardiovascular diseases (14, 15). It also increases health care costs in related areas (16).

In previous studies, respiratory disease, cardiovascular disease, and lung disease were typically used as the health endpoints of health risk assessment (14–17). In some studies, asthma, acute bronchitis and chronic bronchitis are also part of the evaluation system (7, 18, 19). Some scholars use country’s air quality standards as health guidelines (20). In other studies, the World Health Organization (WHO) air quality guidelines are generally used as the baseline concentration for calculation. No matter which standard is adopted, it reflects people’s concern for environmental safety. That focus has been growing in recent years.

In March 2021, the “14th Five-Year Plan for National Economic and Social Development of the People’s Republic of China and the Outline of Long-term Goals for 2035” offered to intensify the battle against pollution and basically eliminate heavy pollution days. In October 2021, 10 ministries and commissions including the Ministry of Ecology and Environment and the governments of seven provinces (municipalities) including Shandong jointly issued the “Plan for Comprehensive Control of Air Pollution in Autumn and Winter 2021–2022.” 13 of the 16 cities in Shandong were included in the strategic control regions. In the “Action Plan for the Treatment of New Pollutants (2022)” issued by the General Office of the State Council, environmental health risk prevention has also been put at the heart of the case. Reducing the health risks and costs of PM_2.5_ pollution is a growing concern. As one of the most polluted areas in North China, Shandong is still facing a severe situation of air pollution prevention and control (21, 22). And the health and economic costs caused by PM_2.5_ pollution in Shandong should be made seriously.

As the third largest province in GDP in China, Shandong was plagued by air pollution (23). Although air quality in Shandong had been improving in recent years, heavy pollution events were still common in some cities (24–26). At present, only a few developed cites in Shandong have publicly reported the health risk of PM_2.5_, such as Jinan and Qingdao (27, 28). There was not any accurate data on the health cost of PM_2.5_ pollution for the whole Shandong Province. According to the relevant studies in key regions such as Beijing-Tianjin-Hebei, Yangtze River Delta and Pearl River Delta, the health cost of PM_2.5_ pollution exposure might accounts for 0.3–1.0% of the total annual GDP (18, 29–33). In 2021, the health cost caused by PM_2.5_ pollution in Shandong Province was preliminarily estimated to be about 3.86–12.88 billion dollars. On September 22, 2021, the WHO further improved the original air quality guidelines based on the conclusions of the current important reports by global scholars, and lowered the annual recommended level of PM_2.5_ from 10 μg/m^3^ to 5 μg/m^3^. The 24-h recommended level of PM_2.5_ was reduced from 25 μg/m^3^ to 15 μg/m^3^. The reduction in the health guideline concentration means a change in the original health risk assessment criteria for PM_2.5_ exposure. It also implies that the economic cost of PM_2.5_ exposure may have been underestimated.

In order to understand the PM_2.5_ health risk in Shandong Province. In this study, the health and economic effects of PM_2.5_ exposure in Shandong were evaluated using the new WHO guidelines as health threshold. The evaluation results were also compared with our previous study to discuss the impact of the new WHO guidelines on health risk assessment. Finally, the prevention and control strategies of air pollution in China were discussed based on the evaluation results. Therefore, this study will help clarify the health costs of PM_2.5_ pollution and fill the gap on the health economic effects of PM_2.5_ pollution in Shandong Province. It also provided scientific reference for the optimization of air pollution control strategy in China.

## Materials and methods

### Location information

**Figure 1:**
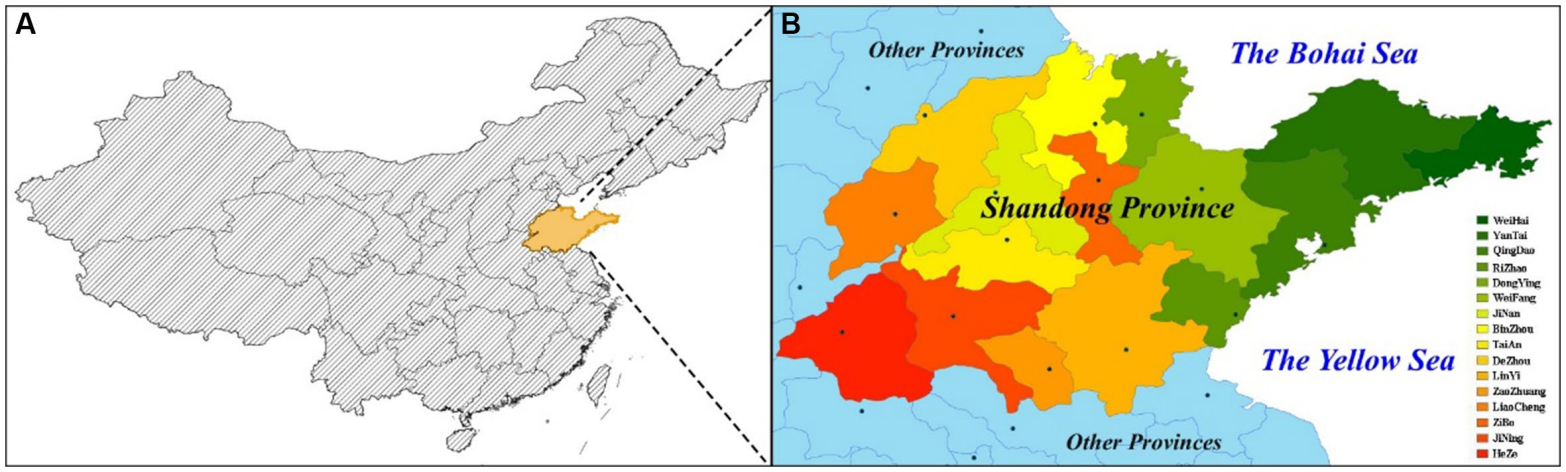
**(A)** Shandong Province within China; **(B)** Cities in Shandong Province.

Shandong Province is situated in the North China Plain, on the east coast of China. It consists of 16 cities (Figure 1). It covers an area of 158,000 square kilometers and has a population of over 101.5 millions (2021). Basic data in 16 cities of Shandong Province was shown in Table 1. The annual average concentration of PM_2.5_ was 39 μg/m^3^, a year-on-year improvement of 15.2% (2021). The annual average concentration of 39 μg/m^3^ was well above the new health guidelines of WHO. The Ambient Air Quality Composite Index, which takes into account the concentrations of six pollutants including PM_2.5_, PM_10_, SO_2_, NO_2_, CO, and O_3_, is used to rank the air quality of 168 key Chinese cities. In this comprehensive index ranking of 168 key cities in China, four cities including Zibo, Liaocheng, Heze and Zaozhuang were in the bottom 20. The situation of air pollution prevention and control in Shandong province was still serious.

**Table 1:** Basic data in 16 cities of Shandong Province.

Districts	PM_2.5_ annual average concentration (μg/m^3^)	Residents (million people)	Male/female ratio	Area (Km^2^)
WeiHai	24	2.907	0.978	5,799
YanTai	27	7.102	0.985	13,864
QingDao	28	10.072	0.973	11,293
Rizhao	31	2.968	1.034	5,358
DongYing	36	2.194	0.986	8,243
WeiFang	38	9.387	1.015	16,167
JiNan	40	9.202	0.982	10,244
BinZhou	40	3.929	1.018	9,660
TaiAn	42	5.472	1.020	7,762
DeZhou	42	5.611	1.028	10,356
LinYi	43	11.018	1.071	17,191
ZaoZhuang	45	3.856	1.098	4,564
LiaoCheng	46	5.952	1.058	8,628
ZiBo	47	4.704	0.985	5,965
JiNing	47	8.358	1.064	11,187
HeZe	48	8.796	1.093	12,239

### PM_2.5_ concentration data

Shandong is one of the provinces with serious air pollution in north China, especially in the western part of Shandong. According to the Bulletin of Ecological Environment of Shandong Province (BEESP 2021), only four cities had PM_2.5_ concentrations that met the II-level National Guidance Standard, including Qingdao, Yantai, Weihai, and Rizhao. The remaining 12 cities had average annual concentrations of more than 35 μg/m^3^. All of the 16 cities failed to meet WHO health guideline. In this study, the PM_2.5_ data was obtained from the Bulletin of Ecological Environment of Shandong Province (BEESP 2021),^1^ Shandong Environmental Air Quality Status Report (SEAQSR 2021),^2^ and the Official website of Shandong Department of Ecology and Environment.^3^

### Population health information

Since population health data were difficult to obtain, the health data used in this study mainly came from the Disease and Health Status Report of Residents in Shandong Province (DHSR 2016; it can be obtained by contacting corresponding author) and the Report on Incidence and Mortality of Key Chronic Diseases in Shandong Province (RIMKCD 2018; it can be obtained by contacting corresponding author). The health cost data was obtained from the Statistical Bulletin of Health Development of Shandong Province (SBHDSP 2021).^4^ Population data were obtained from the Seventh National Census (SNC 2021)^5^ published in May 2021. This study also assessed the health risks of PM_2.5_ for different genders in Shandong. The Male/Female ratio was from the public security household registration statistics in Shandong Statistical Yearbook (SSY 2022).^6^ Area data was drawn from government portals.

### PM_2.5_ health effect assessment

To assess the health risks of PM_2.5_ exposure, the first step should be to correlate PM_2.5_ concentrations with population health. Therefore, it is a critical step to determine the exposure response coefficients used in this work. In this study, all exposure response coefficients were referenced from our earlier studies and other recent relevant studies in China (18, 27, 34–36). Table 2 presents the baseline incidence for six health endpoints in Shandong Province.

**Table 2:** Baseline incidences and exposure-response coefficients associated with 10 μg m^−3^ increment of PM_2.5_.

Health endpoints	Incidence			Coefficients *β_i_* (95% CI)	References
	Male	Female	All		
All-cause mortality	0.0081804	0.0064967	0.0073556	0.0090 (0, 0.0180) (35)	RIMKCD (2018)
Cardiovascular mortality	0.0021664	0.0022355	0.0022003	0.0053 (0.0085, 0.0201) (34)	RIMKCD (2018)
Respiratory mortality	0.0006453	0.0005697	0.0006078	0.0143 (0.0085, 0.0201) (34)	DHSR (2016)
Lung-cancer mortality	0.0008456	0.0004147	0.0006345	0.0340 (0, 0.0710) (34)	RIMKCD (2018)
Cardiovascular hospital admission	0.0154545	0.0185454	0.017	0.0068 (0.0043, 0.0093) (18)	CHSY (2021)
Respiratory hospital admission	0.0199091	0.0238909	0.0219	0.0109 (0, 0.0221) (18)	CHSY (2021)
Lung-cancer morbidity	0.0009505	0.0005583	0.0007554	0.0340 (0, 0.0710) (27)	RIMKCD (2018)

In a large population, the occurrence of disease can be regarded as a low probability event (18, 27). Therefore, its probability of occurrence should conform to the Poisson distribution (34). In this study, the health risk was calculated with the PM exposure response function which was the WHO recommended model for health effect estimation in high PM concentration area (WHO, 2006). There are four major factors in the Equations (1) and (2), which including population size, PM_2.5_ concentration, exposure response coefficient, and the baseline incidence of health endpoint.


(1) $$E_{i}=E_{0}\exp\left[\beta_{i}\left(C-C_{0}\right)\right]$$

(2) $$\Delta E=P\left(E_{i}-E_{0}\right)=P\left\{1-\frac{1}{\exp\left[\beta_{i}\left(C-C_{0}\right)\right]}\right\}E_{i}$$

Here, *E*_0_ is baseline incidence of a disease, *E*_i_ is the estimated incidence of health endpoint *i* under PM_2.5_ concentration *C*, *C*_0_ is the baseline concentration of PM_2.5_ (set as 5 μg/m^3^, the new WHO annual guideline concentration), *C* is the exposure concentration of PM_2.5_, *β_i_* is the exposure response coefficient. *P* is the population size, *∆E* is for population health risks associated with PM_2.5_ pollution. In this study, *C*_0_ refers to the new WHO standards.

### PM_2.5_ economic effect assessment

In this study, health economic losses were estimated using health risk assessment results and average disease costs. The economic effect of PM_2.5_ was assessed with the following equation:


(3) $${\mathrm{EC}}_{i}=\Delta E\cdot \cos t_{i}$$

where EC*_i_* is the total cost of health endpoint *i*; Cost*_i_* is the cost per case.

Here, the health economic effect of hospitalization was estimated using the cost of illness (COI) method (7, 18, 27). Hospitalization costs were obtained from the SBHDSP 2021. Premature death cost was estimated using the method of value of statistical life (VSL) (37). VSL refers to the willingness-to-pay of patients to avoid risk of death. Since willingness-to-pay usually increases with people’s income, a adjusted equation was utilized to correct VSL in this study (27). The *per capita* income was obtained from Shandong Statistical Yearbook. The adjusted equation of VSL as following:


(4) $${\mathrm{VSL}}_{\mathrm{now}}={\mathrm{VSL}}_{\mathrm{past}}{\left(\frac{{\mathrm{Income}}_{\mathrm{now}}}{{\mathrm{Income}}_{\mathrm{past}}}\right)}^{e}$$

where VSL_now_ and VSL_past_ refers to current and past willingness to pay; Income_now_ and Income_past_ represents current and past *per capita* income; *e* is an elastic coefficient of willingness-to-pay assumed to be 0.8. In this study, VSL was adjusted twice because it lacked a reliable reference value in Shandong. Firstly, it was adjusted to get VSL_2021_ based on VSL_2016_ in Jinan. Then, it was adjusted again to get VSL_Shandong_ based on the VSL_Jinan_ in 2021.

## Results and discussion

### PM_2.5_ concentration status report

**Figure 2:**
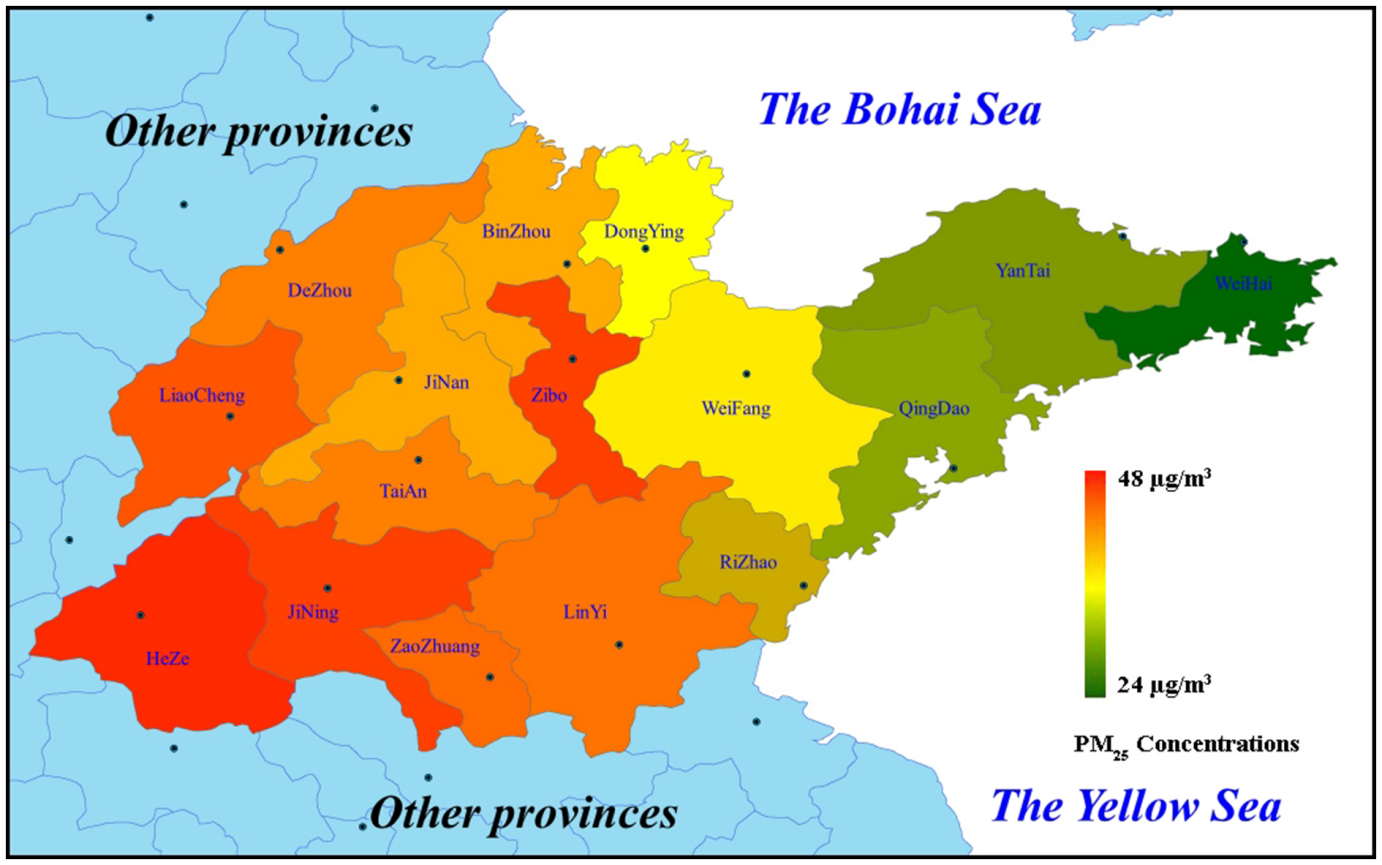
PM_2.5_ concentrations in 16 cities of Shandong Province in 2021.

Pollutant concentration is one of the important factors affecting health risk assessment results (38–40). High levels of PM_2.5_ exposure will increase the risk of some health endpoints such as respiratory, cardiovascular and lung diseases (41–43). As shown in Figure 2, the PM_2.5_ concentration was relatively low in the area of Shandong Peninsula. While it had a high concentration in the western area of Shandong province. Industrial distribution and regional differences, as well as unbalanced economic development, might lead to the spatial differences in PM_2.5_ concentration in Shandong. The PM_2.5_ concentrations of 16 cities in Shandong Province have been provided in Table 1. Therefore, the health effects of PM_2.5_ pollution were likely to be greater in the western area of Shandong province without considering the influence of population density factor. PM_2.5_ pollution might have great impact on the four cities including Heze, Jining, Zibo, and Liaocheng.

### PM_2.5_ health risk assessment

As shown in Table 2, the baseline incidences of health endpoints were provided. The incidences of cardiovascular and respiratory diseases were obtained from China Health Statistics Yearbook (CHSY, 2021)^7^ Male/Female incidences were calculated based on the hospitalization rate of residents and the sex ratio of hospitalized patients in the national survey data in 2018. Due to the exposure-response coefficient of lung cancer morbidity cannot be obtained, its coefficient referred to lung-cancer mortality in this work.

The evaluation result showed that the premature death related to PM_2.5_ pollution contributed 3.16% of all-cause deaths (shown in Table 3). Among them, the proportion of male was 1.79%, and the proportion of female was 1.37%. Cardiovascular mortality, respiratory mortality, and lung-cancer mortality related to PM_2.5_ pollution contributed 1.85, 5.08, and 12.51%, respectively, to annual cases of these health endpoints. Cardiovascular hospital admission, respiratory hospital admission, and lung-cancer morbidity related to PM_2.5_ pollution contributed 2.38, 3.85, and 12.51% to yearly cases of these health endpoints. The four health endpoints related to PM_2.5_ pollution including all-cause mortality, respiratory mortality, lung-cancer mortality, and lung-cancer morbidity in male were higher than those in female. The contribution of cardiovascular mortality in male and female was roughly equal. The contributions of cardiovascular and respiratory hospital admission in female were higher than those in male. This result was consistent with the findings reported by Bell et al. (44) and Sang et al. (45). Bell et al. (44) pointed out that women might be more susceptible to PM_2.5_-related hospitalizations for some respiratory and cardiovascular causes. Sang et al. (45) suggested that global ambient PM_2.5_ pollution caused more premature deaths and consumption in men than in women. Therefore, PM_2.5_ pollution had a greater impact on respiratory mortality and lung-cancer mortality and morbidity in male. And it also made a significant contribution to all-cause premature deaths in male. While it played an import role on cardiovascular and respiratory hospital admission in female. As a whole, the health consequences of PM_2.5_ pollution appeared to be more severe in male than in female. For male, more attention should be paid to daily physical examination to reduce the premature death risk from diseases related to PM_2.5_ pollution, especially respiratory system examination including lungs and respiratory tract.

**Table 3:** Health effect of PM_2.5_ in Shandong in 2021.

Gender	Health endpoints	WeiHai	YanTai	QingDao	Rizhao	DongYing	WeiFang	JiNan	BinZhou	TaiAn	DeZhou	LinYi	ZaoZhuang	LiaoCheng	ZiBo	JiNing	HeZe	Sum.	Annual %**
Male	All-cause mortality	203	577	850	292	252	1,166	1,194	519	765	788	1,622	605	941	736	1,358	1,483	13,349	1.79
	*Cardiovascular mortality*	*32*	*90*	*132*	*45*	*39*	*181*	*185*	*80*	*119*	*122*	*251*	*94*	*146*	*114*	*210*	*229*	*2,068*	*0.93*
	*Respiratory mortality*	*26*	*73*	*107*	*37*	*32*	*147*	*151*	*66*	*97*	*100*	*205*	*77*	*119*	*93*	*172*	*188*	*1,689*	*2.74*
	*Lung-cancer mortality*	*81*	*231*	*342*	*118*	*102*	*475*	*487*	*212*	*313*	*322*	*665*	*249*	*387*	*303*	*559*	*611*	*5,458*	*8.47*
	Cardiovascular hospital admission	289	821	1,210	416	359	1,658	1,697	738	1,088	1,120	2,305	860	1,337	1,045	1,929	2,106	18,978	1.10
	Respiratory hospital admission	599	1,703	2,511	863	745	3,448	3,530	1,534	2,264	2,330	4,797	1,790	2,784	2,177	4,018	4,388	39,483	1.78
	Lung-cancer morbidity	91	260	384	133	115	534	548	238	352	362	747	279	435	341	629	687	6,135	8.00
Female	All-cause mortality	165	465	694	224	203	912	965	405	596	609	1,203	438	706	593	1,013	1,077	10,268	1.37
	*Cardiovascular mortality*	*33*	*94*	*140*	*45*	*41*	*184*	*194*	*81*	*120*	*122*	*242*	*88*	*142*	*119*	*204*	*217*	*2,067*	*0.93*
	*Respiratory mortality*	*23*	*65*	*97*	*31*	*29*	*128*	*136*	*57*	*84*	*86*	*169*	*62*	*99*	*84*	*143*	*152*	*1,444*	*2.34*
	*Lung-cancer mortality*	*41*	*115*	*172*	*56*	*51*	*229*	*243*	*102*	*151*	*154*	*304*	*111*	*179*	*151*	*258*	*274*	*2,592*	*4.02*
	Cardiovascular hospital admission	354	1,000	1,492	483	436	1,961	2,074	870	1,280	1,307	2,583	940	1,516	1,273	2,176	2,313	22,058	1.28
	Respiratory hospital admission	735	2,075	3,096	1,002	907	4,076	4,313	1,809	2,663	2,720	5,375	1,957	3,158	2,652	4,532	4,818	45,889	2.06
	Lung-cancer morbidity	55	155	232	75	69	309	328	137	203	207	410	149	242	203	347	369	3,489	4.55
All	All-cause mortality	369	1,045	1,550	517	457	2,081	2,166	925	1,363	1,398	2,820	1,040	1,646	1,333	2,368	2,553	23,628	3.16
	*Cardiovascular mortality*	*65*	*183*	*272*	*91*	*80*	*364*	*379*	*162*	*238*	*244*	*493*	*182*	*288*	*233*	*414*	*446*	*4,134*	*1.85*
	*Respiratory mortality*	*49*	*138*	*205*	*68*	*60*	*276*	*287*	*123*	*181*	*185*	*374*	*138*	*218*	*177*	*314*	*339*	*3,132*	*5.08*
	*Lung-cancer mortality*	*123*	*350*	*520*	*174*	*155*	*707*	*738*	*315*	*465*	*477*	*964*	*356*	*565*	*458*	*814*	*879*	*8,061*	*12.51*
	Cardiovascular hospital admission	643	1,820	2,699	900	795	3,621	3,768	1,609	2,370	2,430	4,903	1,807	2,861	2,317	4,116	4,437	41,096	2.38
	Respiratory hospital admission	1,332	3,775	5,600	1,868	1,651	7,529	7,837	3,346	4,932	5,057	10,204	3,763	5,957	4,826	8,574	9,244	85,496	3.85
	Lung-cancer morbidity	147	417	619	207	184	842	878	375	554	568	1,148	424	673	545	969	1,046	9,597	12.51
	Sum.*	2,490	7,056	10,467	3,493	3,087	14,074	14,649	6,255	9,219	9,453	19,075	7,035	11,136	9,021	16,028	17,279	159,817	/

Male + Female≠All. Baseline incidence was calculated separately, which caused this error (<3%).

*Sum = *All cause mortality + Respiratory hospital admission + Cardiovascular hospital admission + Lung-cancer morbidity*.

**Cases caused by PM_2.5_/annual cases of this health endpoint.

Italics: All-cause mortality including Cardiovascular mortality, Respiratory mortality, and Lung-cancer mortality.

Based on the evaluation results of this study, the number of premature deaths and illnesses related to PM_2.5_ pollution in Shandong Province reached 159,817 in 2021. Without considering population density, LinYi, HeZe, JiNing, JiNan and WeiFang had higher health risks caused by PM_2.5_ pollution. In each of these cities, more than 14,000 people experienced premature death or morbidity due to PM_2.5_ pollution. Only three cities, WeiHai, DongYing and Rizhao, were less impacted by PM_2.5_ pollution in terms of health risk. The number of premature deaths and illnesses affected by PM_2.5_ in each of these areas was less than 5,000. Therefore, further strengthening the control of PM_2.5_ emission will have a positive effect on population health, especially in areas with high health risks.

### PM_2.5_ health economic costs

Health economic effect assessment is an important means to evaluate the economic burden of environmental pollution to a city (46–49). The value of statistical life (VSL) method was a common method to assess the health cost of premature death in previous studies (34). The occurrence of respiratory and cardiovascular diseases is closely related to PM pollution, which has been confirmed in many previous studies (50–54). Therefore, the hospitalization costs for respiratory and cardiovascular diseases were also assessed in addition to premature death endpoint in this study. Since the exact cost of each disease could not be obtained, the mean hospitalization cost was selected as the reference value for calculation in this work. Owing to the high mortality rate of lung-cancer, its health cost was estimated using VSL method in this study. The costs of premature death and hospitalization were shown in Table 4. Finally, the economic effect related to PM_2.5_ pollution was assessed based on the result of health risk assessment.

**Table 4:** Health cost situation.

Health endpoints	Costs (US$)				Approach	References
	Hospital	Community health center	Town and township hospital	Mean		
Mortality	/	/	/	219,000	VSL	Yin et al. (35)
Hospital admission	1,753	607	450	937	COI	SBHDSP (2021)

Some scholars suggested that the health economic costs caused by PM_2.5_ pollution could be around 1% of GDP (7, 22). As shown in Table 5, the health economic loss of each heath endpoint related to PM_2.5_ pollution was estimated. It resulted in nearly 7.4 billions dollars in healthy economic cost, which accounted for 0.57% of GDP in Shandong in 2021. This result was basically consistent with our previous study in Beijing. It accounted for 0.87, 0.54, and 0.45% of GDP in Beijing during 2014–2016, respectively (34). The percentage of health economic loss in GDP was lower than other long-term exposure studies in China (18, 27, 35). It may be due to the failure to account for outpatient costs, such as asthma, acute bronchitis and chronic bronchitis. In addition, the reduction of air pollution in China may also be a factor in the falling economic costs of health (34, 55, 56).

**Table 5:** Health economic effect of PM_2.5_ exposure in Shandong in 2021 (million US$).

Gender	Health endpoints	WeiHai	YanTai	QingDao	Rizhao	DongYing	WeiFang	JiNan	BinZhou	TaiAn	DeZhou	LinYi	ZaoZhuang	LiaoCheng	ZiBo	JiNing	HeZe	Sum.	EC_i_/GDP (%)**
Male	All-cause mortality	44.5	126.4	186.2	63.9	55.2	255.4	261.5	113.7	167.5	172.6	355.2	132.5	206.1	161.2	297.4	324.8	2923.9	0.227
	*Cardiovascular mortality*	*7.0*	*19.7*	*28.9*	*9.9*	*8.5*	*39.6*	*40.5*	*17.5*	*26.1*	*26.7*	*55.0*	*20.6*	*32.0*	*25.0*	*46.0*	*50.2*	*453.1*	*0.035*
	*Respiratory mortality*	*5.7*	*16.0*	*23.4*	*8.1*	*7.0*	*32.2*	*33.1*	*14.5*	*21.2*	*21.9*	*44.9*	*16.9*	*26.1*	*20.4*	*37.7*	*41.2*	*370.1*	*0.029*
	*Lung-cancer mortality*	*17.7*	*50.6*	*74.9*	*25.8*	*22.3*	*104.0*	*106.7*	*46.4*	*68.5*	*70.5*	*145.6*	*54.5*	*84.8*	*66.4*	*122.4*	*133.8*	*1195.1*	*0.093*
	Cardiovascular hospital admission	0.3	0.8	1.1	0.4	0.3	1.6	1.6	0.7	1.0	1.0	2.2	0.8	1.3	1.0	1.8	2.0	17.8	0.001
	Respiratory hospital admission	0.6	1.6	2.4	0.8	0.7	3.2	3.3	1.4	2.1	2.2	4.5	1.7	2.6	2.0	3.8	4.1	37.0	0.003
	Lung-cancer morbidity	19.9	56.9	84.1	29.1	25.2	116.9	120.0	52.1	77.1	79.3	163.6	61.1	95.3	74.7	137.8	150.5	1343.6	0.104
Female	All-cause mortality	36.1	101.8	152.0	49.1	44.5	199.7	211.3	88.7	130.5	133.4	263.5	95.9	154.6	129.9	221.8	235.9	2248.7	0.175
	*Cardiovascular mortality*	*7.2*	*20.6*	*30.7*	*9.9*	*9.0*	*40.3*	*42.5*	*17.7*	*26.3*	*26.7*	*53.0*	*19.3*	*31.1*	*26.1*	*44.7*	*47.5*	*452.5*	*0.035*
	*Respiratory mortality*	*5.0*	*14.2*	*21.2*	*6.8*	*6.4*	*28.0*	*29.8*	*12.5*	*18.4*	*18.8*	*37.0*	*13.6*	*21.7*	*18.4*	*31.3*	*33.3*	*316.5*	*0.025*
	*Lung-cancer mortality*	*9.0*	*25.2*	*37.7*	*12.3*	*11.2*	*50.2*	*53.2*	*22.3*	*33.1*	*33.7*	*66.6*	*24.3*	*39.2*	*33.1*	*56.5*	*60.0*	*567.4*	*0.044*
	Cardiovascular hospital admission	0.3	0.9	1.4	0.5	0.4	1.8	1.9	0.8	1.2	1.2	2.4	0.9	1.4	1.2	2.0	2.2	20.7	0.002
	Respiratory hospital admission	0.7	1.9	2.9	0.9	0.8	3.8	4.0	1.7	2.5	2.5	5.0	1.8	3.0	2.5	4.2	4.5	43.0	0.003
	Lung-cancer morbidity	12.0	33.9	50.8	16.4	15.1	67.7	71.8	30.0	44.5	45.3	89.8	32.6	53.0	44.5	76.0	80.8	764.3	0.059
All	All-cause mortality	80.8	228.9	339.5	113.2	100.1	455.7	474.4	202.6	298.5	306.2	617.6	227.8	360.5	291.9	518.6	559.1	5175.2	0.402
	*Cardiovascular mortality*	*14.2*	*40.1*	*59.6*	*19.9*	*17.5*	*79.7*	*83.0*	*35.5*	*52.1*	*53.4*	*108.0*	*39.9*	*63.1*	*51.0*	*90.7*	*97.7*	*905.3*	*0.070*
	*Respiratory mortality*	*10.7*	*30.2*	*44.9*	*14.9*	*13.1*	*60.4*	*62.9*	*26.9*	*39.6*	*40.5*	*81.9*	*30.2*	*47.7*	*38.8*	*68.8*	*74.2*	*685.9*	*0.053*
	*Lung-cancer mortality*	*26.9*	*76.7*	*113.9*	*38.1*	*33.9*	*154.8*	*161.6*	*69.0*	*101.8*	*104.5*	*211.1*	*78.0*	*123.7*	*100.3*	*178.3*	*192.5*	*1765.1*	*0.137*
	Cardiovascular hospital admission	0.6	1.7	2.5	0.8	0.7	3.4	3.5	1.5	2.2	2.3	4.6	1.7	2.7	2.2	3.9	4.2	38.5	0.003
	Respiratory hospital admission	1.2	3.5	5.2	1.8	1.5	7.1	7.3	3.1	4.6	4.7	9.6	3.5	5.6	4.5	8.0	8.7	80.1	0.006
	Lung-cancer morbidity	32.2	91.3	135.6	45.3	40.3	184.4	192.3	82.1	121.3	124.4	251.4	92.9	147.4	119.4	212.2	229.1	2101.5	0.163
	Sum.*	114.9	325.4	482.8	161.1	142.7	650.6	677.5	289.3	426.7	437.6	883.1	325.8	516.1	418.0	742.7	801.0	7395.3	0.574
	EC_city_/GDP_city_(%)#	0.21	0.24	0.22	0.47	0.27	0.60	0.38	0.65	0.92	0.81	1.04	1.08	1.26	0.64	0.95	1.30	0.57	/

*Sum = All cause mortality + Respiratory hospital admission + Cardiovascular hospital admission + Lung-cancer morbidity.

**Economic costs caused by health endpoint *i*/the GDP in Shandong in 2021.

^#^ Economic costs caused by PM2.5/the local GDP in 2021.

Italics: All-cause mortality including Cardiovascular mortality, Respiratory mortality, and Lung-cancer mortality.

Overall, the health economic effects were higher for male than for female in Shandong. The health economic costs of male and female accounted for 0.336 and 0.239% of GDP, respectively. In terms of the health effects in cities, LinYi, HeZe, JiNing, JiNan, WeiFang, and LiaoCheng were the cities where the health economic cost was more than 500 millions. For the proportion of health economic cost, HeZe, LiaoCheng, ZaoZhuang, and LinYi were the cities where it was more than 1% of GDP, accounted for 1.30, 1.26, 1.08, and 1.04% of the GDP in local areas. On the whole, the economic cost of health in highly polluted and densely populated areas in Shandong was higher than that in other cities. It also led to a heavier fiscal burden for these areas.

### Policies implication

Since the publication of the WHO Air Quality Guidelines - Global Update 2005 (AQG2005), it has had a positive impact on air pollution control policies around the world (57, 58). AQG2005 provided the first globally referenced framework for air pollution control targets and established transitional targets based on the potential risk of death from long-term exposure to each pollutant (59–61). It was then adopted by many highly polluted regions and countries as progressive targets for the gradual reduction of air pollution (62). China also updated its Air Quality Standards in 2012, and included PM_2.5_ and O_3_ in monitoring projects for the first time (63). With the progress of science, the monitoring capabilities of environmental and health and the level of exposure and risk assessment had gradually improved (64). It led a significant increase in scientific evidence of the health hazards of air pollution (65, 66). Finally, WHO updated the AQG again in September 2021 on the basis of comprehensive analysis and scientific assessment of the literature and results over the past 15 years. Air quality standards have become more stringent.

As air quality standards have been ever more stringent, PM_2.5_ health guideline has also been changed and further reduced. Population health risks and economic effects assessed based on the new WHO standards should be higher than that using the previous air quality standards. However, the increase of health risks and economic costs related to PM_2.5_ pollution was not very significant compared with our previous study. Considering these differences in population, economy, and environment, making a direct comparison between Shandong and Beijing may not be entirely appropriate. The total health effects and economic losses caused by PM_2.5_ pollution may vary greatly in the two regions. Therefore, in order to reduce the uncertain impact of these factors, this study only compared the proportion of PM_2.5_ pollution-related health endpoints and the proportion of economic loss in local GDP between the two regions. Finally, whether it was the proportion of affected population or the proportion of health economic costs, the results of this study were comparable to our previous assessment of Beijing in 2015 (34). In our previous study of Beijing, it was the Class II limit values of the National Ambient Air Quality Standard (35 μg/m^3^) that used as the baseline concentration to complete the health risk assessment work. It was a full 30 μg/m^3^ higher than the baseline concentration used in this study. The fact that the health and economic effects related to PM_2.5_ did not increase significantly under the stricter standards can only be attributed to the possibility that China’s air pollution control measures were having a positive effect. The annual PM_2.5_ concentration assessed in this study should be at least 30 μg/m^3^ lower than that in Beijing in 2015. In fact, the PM_2.5_ concentration in Beijing was 80.6 μg/m^3^ in 2015, while it was 39 μg/m^3^ in Shandong in 2021. Therefore, with the positive effect of China’s air pollution control measures, the nationwide decrease in PM_2.5_ concentration was the main reason why the health and economic effects related to PM_2.5_ pollution had not increased substantially in this study. China’s air quality improvement strategy had started to pay off, which was confirmed in this study from the perspective of health risk assessment.

Although the results of this study were mainly based on the analysis of PM_2.5_ pollution in Shandong Province, they still provided side evidence for the positive effects of air quality improvement strategies in China. In the follow-up studies, strengthening regional difference analysis and long-term assessment may be more valuable for evaluating China’s air quality prevention and control strategies. In addition, how to tailor the prevention and control strategies of different regions according to the health risks of regional populations should also attract the attention of decision-making departments. Reducing population health risks should be the ultimate goal of improving air quality.

## Conclusion

In this study, the exposure response function was used to assess the health risks of PM_2.5_ pollution in Shandong Province. The cost of illness (COI) method and value of statistical life (VSL) method were used to estimate the health economic losses associated with PM_2.5_ pollution. The new WHO (2021) Health Guidelines were used as the PM_2.5_ baseline concentration in this study. The health risks and economic effects of PM_2.5_ exposure in 16 cities in Shandong Province were assessed separately. Results showed that despite a 30 μg/m^3^ reduction in PM_2.5_ baseline concentration compared to our previous study, there was no significant increase in health risks and economic losses. About 159.8 thousand people died or became ill prematurely due to PM_2.5_ pollution, which caused a health economic loss of about 7.4 billion dollars in Shandong. The health economic cost accounted for about 0.57% of GDP in Shandong in 2021. It was similar to our previous assessment of the economic effects of PM_2.5_ pollution in Beijing in 2015. Therefore, under the more stringent criteria, there was no qualitative change in the assessment of health risks and economic losses, which proved that China’s air pollution prevention and control strategy might already be having a positive effect.

## Data availability statement

The original contributions presented in the study are included in the article/supplementary material, further inquiries can be directed to the corresponding authors.

## Author contributions

XX: Writing – review and editing, Conceptualization, Methodology, Project administration. WZ: Writing – review and editing. XS: Review and editing. ZS: Writing – review and editing, Formal Analysis. WC: Review and editing. YW: Writing – review and editing. HM: Writing – review and editing. TL: Writing – review and editing. ZW: Review and editing. All authors contributed to the article and approved the submitted version.

## Footnotes

^1^ http://xxgk.sdein.gov.cn/xxgkml/hjzkgb/202206/P020220607364593369389.pdf

^2^ http://www.shandong.gov.cn/art/2022/1/24/art_305267_10331968.html?xxgkhide=1

^3^ http://sthj.shandong.gov.cn/zwgk/sqcspm/

^4^ http://www.shandong.gov.cn/art/2023/1/10/art_305258_10333917.html

^5^ http://tjj.shandong.gov.cn/art/2021/5/21/art_156112_10287516.html

^6^ http://tjj.shandong.gov.cn/tjnj/nj2022/zk/zk/indexch.htm

^7^ https://www.doc88.com/p-11461558491027.html
